# Frequency of fish/seafood consumption and risk of stroke: a prospective cohort study in Zhejiang, China

**DOI:** 10.1186/s12986-026-01136-x

**Published:** 2026-05-11

**Authors:** Hao Wang, Huaidong Du, Lingli Chen, Kaixu Xie, Zhengjie Shen, Chen Chen, Haidong Xu, Jun Lv, Canqing Yu, Pei Pei, Dianjianyi Sun, Min Yu, Jieming Zhong

**Affiliations:** 1 Department of Non-Communicable Diseases Control and Prevention, Zhejiang Provincial Center for Disease Control and Prevention, Hangzhou, 310051 China; 2 Clinical Trial Service Unit & Epidemiological Studies Unit, Nuffield Department of Population Health, University of Oxford, Oxford, OX3 7LF UK; 3 Department of Non-Communicable Diseases Control and Prevention, Tongxiang City Center for Disease Control and Prevention, Tongxiang, 314599 China; 4 Department of Epidemiology & Biostatistics, School of Public Health, Peking University, Beijing, 100083 China; 5 Peking University Center for Public Health and Epidemic Preparedness and Response, Beijing, 100083 China; 6 Key Laboratory of Epidemiology of Major Diseases (Peking University), Ministry of Education, Beijing, 100083 China

**Keywords:** Fish consumption, Seafood consumption, Stroke, Prospective study, China

## Abstract

**Background:**

The association of fish/seafood consumption with stroke remains inconclusive.

**Methods:**

This cohort included 53,916 participants aged 30–79 years from the China Kadoorie Biobank prospective study in Tongxiang. Incident stroke was obtained periodically through linkage with stroke surveillance system, death registries, and national health insurance system. Cox regressions were used to estimate the association between the frequency of fish/seafood consumption and incident stroke or its subtypes.

**Results:**

48.2% of participants reported consuming fish/seafood at least once per week. During 643,100 person-years (median 12.4 years) of follow-up, 2994 total strokes, 2213 ischemic strokes (IS), and 772 hemorrhagic strokes (HS) were identified. Among all participants, fish/seafood consumption was not associated with the risk of total stroke or its subtypes. Among men, in comparison with non-consumers, the adjusted HRs (95%CI) for total stroke risk were 0.90 (0.74–1.11) for monthly, 0.83 (0.68–1.03) for 1–3 days/week, and 0.64 (0.45–0.91) for ≥ 4 days/week consumption (*p*-trend = 0.009). A similar protective pattern was observed for IS (*p*-trend = 0.024), whereas no association was observed with HS. Among women, no significant association was observed between fish/seafood consumption and total stroke or its subtypes.

**Conclusions:**

Fish/seafood consumption was found to be unrelated to HS. Higher fish/seafood consumption was associated with lower risks of total stroke and IS in men but not in women. Future studies need to validate these subtype- and sex-specific associations.

**Supplementary Information:**

The online version contains supplementary material available at 10.1186/s12986-026-01136-x.

## Background

The number of stroke disability-adjusted life years (DALYs) has increased in the past decades [1]. The Global Burden Disease Study (GBD) indicated that the global prevalent cases of stroke were estimated to be 93.8 million worldwide in 2021 [2]. The corresponding figure for China was 26.3 million, accounting for more than one quarter of the global stroke number. A nationally representative study of 4.2 million Chinese adults aged ≥ 40 years demonstrated that the prevalence of stroke escalated from 2.28% to 2.58% between 2013 and 2019 [3]. Of all stroke cases, 81.9% were ischemic stroke (IS), and 18.1% were hemorrhagic stroke (HS) [6]. Stroke accounts for a major medical and financial burden in China, and the medical cost of hospitalization in 2019 amounted to an estimated Chinese Yuan (CNY) 54.8 billion, with patients bearing approximately CNY 18.3 billion of this expense [4].

Fish/seafood, a rich source of protein, polyunsaturated fatty acids (PUFAs), and essential vitamins [5], constitutes a fundamental component of the Chinese diet, with per capita consumption reaching 23.7 g daily [6]. Over the past decades, numerous studies have focused on the beneficial effects of fish/seafood intake on stroke [7–11]. However, the association of fish intake with stroke, as reported in published studies, remains contradictory. While some cohort studies documented that fish intake was unrelated to stroke risk [12, 13], others observed an inverse association between fish intake and stroke [11, 14]. Furthermore, most previous research focused on Western populations [13, 17], with limited evidence from China, where dietary patterns, cooking methods, and disease patterns differ substantially from those in Western populations [14, 15]. Previous studies among Chinese populations, due to small sample sizes (e.g., 18215 participants in Guangzhou) [16], short follow-up durations (e.g., median 4.6 years in Shanghai and average 7.3 years in another study) [17, 18], and failure to differentiate stroke subtypes [19], were unable to establish clear associations of fish/seafood consumption with stroke. Hence, the purpose of this study was to examine the association between fish/seafood consumption and risk of incident stroke leveraging data from Tongxiang cohort within the China Kadoorie Biobank (CKB).

## Materials and methods

### Study population and design

The data utilized in the present study were derived from CKB study in Tongxiang, Zhejiang Province. CKB study is a nationwide prospective cohort study conducted in 10 geographically defined regions (5 urban and 5 rural) in China. Details on the study design, method, and participant characteristics have been reported elsewhere previously [20–22]. Tongxiang represents one of the five rural regions. Between June 2004 and July 2008, a baseline survey was administered to 57,704 permanent residents aged 30–79 years with no severe disability (i.e., excluding individuals with severe physical disability, mute persons, or the deaf‌). All participants provided written informed consent.

### Assessment of fish/seafood consumption

Dietary information was systematically evaluated through a validated short food frequency questionnaire (FFQ), which demonstrated good reproducibility and relative validity [23]. Frequency of fish consumption was determined through the question “During the past 12 months, about how often did you eat fish or seafood?” Answer options included “Daily”, “4–6 days per week”, “1–3 days per week”, “Monthly”, and “Never or rarely”. Those who chose “Daily” or “4–6 days per week” were consolidated into one group (i.e., ≥ 4 days/week) to achieve sufficient case numbers. Participants who chose the last option were classified as non-consumers.

### Assessment of covariates

Comprehensive covariate data were systematically collected at baseline through a rigorously standardized procedure. The survey was conducted via face-to-face interview by well-trained interviewers using a laptop-based questionnaire to ensure data accuracy and consistency. The questionnaire encompassed sociodemographic characteristics (age, sex, education attainment, and marital status, etc.), behavioral indicators (cigarette smoking, alcohol drinking, physical activity, fresh fruit consumption, meat consumption, and sleep duration, etc.), personal medical history (cancer, stroke, diabetes, and heart attack), and women’s menopausal status. Physical activity was operationalized as cumulative metabolic equivalent hours per day (MET-hours/day) based on the usual type and duration of activities related to work, transportation, housework, and non-sedentary recreation [24].

All physical measurements were conducted by certified personnel using standardized, calibrated equipment. Standing height was measured to the nearest 0.1 cm (without shoes) using a portable stadiometer, and weight was measured to the nearest 0.1 kg using TBF-300 body composition analyzer (Tanita Inc., Tokyo, Japan). Body mass index (BMI) was calculated as weight in kilograms divided by the square of height in meters, and obesity was defined as BMI ≥ 25.0 kg/m^2^ [25]. Blood pressure was measured twice using a UA-779 digital monitor on the unclothed right upper arm (5-minute interval). If the variation of two times systolic blood pressure (SBP) measurement was larger than 10 mmHg, a third measurement was performed, and the final value for analyses was derived from the average of the last two measurements. Prevalent hypertension was defined if any of the following criteria were met: (a) a measured SBP ≥ 140 mmHg, or (b) a measured diastolic blood pressure (DBP) ≥ 90 mmHg, or (c) a prior history of doctor-diagnosed hypertension, or (d) self-reported use of antihypertensive medication.

Non-fasting venous blood samples (10 mL) were collected from participants, with precise documentation of the time elapsed since the last meal. Immediate on-site testing of plasma glucose level was implemented. Random blood glucose (RBG) levels were measured using the SureStep Plus System (Johnson & Johnson, New Brunswick, NJ, USA). Participants without self-reported diabetes history were classified as screen-detected diabetic individuals if either of the following thresholds was met: (a) RBG ≥ 11.1 mmol/l, or (b) fasting blood glucose ≥ 7.0 mmol/l.

**Fig. 1:**
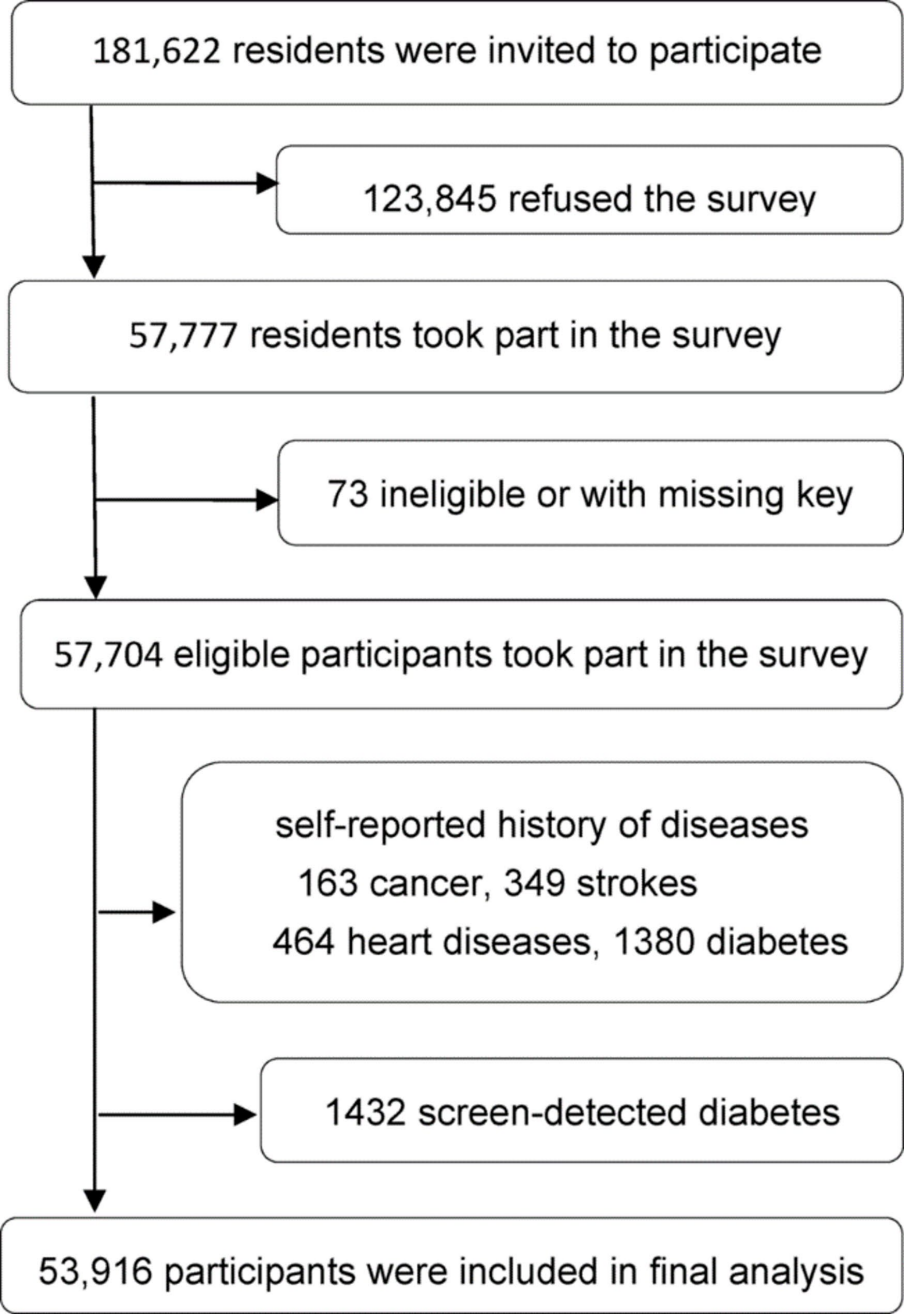
Flowchart of participants included in final analyses

In the present study, participants with a self-reported history of physician-diagnosed cancer (*N* = 163), strokes (*N* = 349), heart diseases (*N* = 464), or diabetes (*N* = 1380) were excluded. In addition, screen-detected diabetic participants (*N* = 1432) were also excluded. Eventually, a total of 53,916 participants, including 22,573 men and 31,343 women, were included in the final analysis. **(**Fig. 1**)**

### Follow-up for the incident stroke

All the participants were prospectively followed from the date of baseline survey until the date of incident stroke, death, or 31 December 2018. Incident strokes were obtained periodically through linkage with the local stroke surveillance system, and death registries. In addition, information on incidence was collected through linkage with the national health insurance claim system (coverage > 99% cohort). Both fatal and non-fatal strokes were coded using the 10th revision of the International Classification of Diseases by trained staff who were blinded to the baseline information. For the present analysis, we included total stroke as I60-I61and I63-I64, IS as I63, and HS as I60-I61.

### Statistical analyses

Percentage and mean values of characteristics at baseline were calculated according to the frequency of fish/seafood consumption. Continuous variables were presented as the mean ± standard deviation (SD), and categorical variables were presented as percentages. Comparing baseline characteristics by groups of fish/seafood consumption, the Mantel-Haenszel chi-square test was used for categorical variables, and Analysis of variance or Kruskal–Wallis rank test was used for continuous variables. Cox proportional hazards regression was constructed to estimate adjusted hazard ratios (HRs) of incident stroke in relation to fish/seafood consumption and 95% confidence intervals. Three different models were performed to calculate HRs of incident stroke risk. In model 1, HRs were adjusted for age, sex, education attainment (no formal education, primary school, middle school, and high school or above), household income (< 19,999 CNY, 20,000–34,999 CNY, ≥ 35,000 CNY), and marital status. In model 2, cigarette smoking (never, occasional, former, and current), alcohol drinking (never, occasional, former, and current), meat consumption (daily and non-daily), fresh fruit consumption (daily and non-daily), physical activity (metabolic equivalent of tasks hours/day; continuous), sleep duration (continuous), and BMI (continuous) were additionally adjusted for. Model 3 included prevalent hypertension status as well. Sex-stratified analyses were performed for men and women separately. In subgroup analyses, participants were combined into two groups (i.e., weekly consumers vs. non-weekly consumers), and whether the associations of weekly fish/seafood consumption with incident total stroke and IS differed according to covariates were detected. Several sensitivity analyses were performed to check the robustness of our findings. HRs were further adjusted for menopause status among women. Fine and Grey’ competing risk model was used to account for the competing risk of all-cause death. Participants with less than two years of follow-up were excluded to reduce the effect of reverse causality. `The proportional hazards assumption was tested via the Schoenfeld residual test. No violations of proportionality were observed. The tests for interaction were performed by means of likelihood ratio tests, which involved comparing models with and without cross-product terms between sex and fish/seafood consumption. All statistical analysis was calculated by using SAS version 9.4, and a two-sided *p* < 0.05 was defined as statistically significant.

## Results

### Characteristics of participants

The mean (standard deviation) baseline age was 52.0 (9.9) years, and 58.1% of participants were female. Of the 53,916 participants included, the proportion of participants who consumed fish/seafood never/rarely, monthly, 1–3 days per week, and ≥ 4 days per week was 7.0%, 44.8%, 45.0%, and 3.2%, respectively. Participants who consumed fish/seafood more frequently were more likely to be younger, men, having a higher level of formal education, wealthy, current smokers, current drinkers, physically inactive, to consume meat and fruit frequently, and having a higher BMI, but less likely to have hypertension (*p* < 0.001). Women who consumed fish/seafood more frequently were less likely to be post-menopausal. **(**Table 1**)**

**Table 1:** Baseline characteristics of the participants according to frequency of fish/seafood consumption

Characteristics	Overall	Frequency of fish/seafood consumption				*p*-value
	(*N* = 53,916) (*N* = 3768)	Never/rarely (*N* = 24,148)	Monthly (*N* = 24,252)	1–3 days/week (*N* = 1748)	≥ 4 days/week	
Mean age (years)	52.0 ± 9.9	54.6 ± 10.4	53.6 ± 10.1	51.2 ± 9.4	50.5 ± 9.4	< 0.001
Women (%)	58.1	69.6	60.8	54.3	49.3	< 0.001
No formal education (%)	43.5	61.9	47.3	37.5	32.3	< 0.001
Income ≥ 35,000 (CNY) (%)	37.8	20.8	34.6	43.0	45.4	< 0.001
Current smokers (%)	28.0	20.5	26.1	30.5	36.0	< 0.001
Current drinkers (%)	17.1	10.3	15.3	19.4	24.4	< 0.001
Physical activities (MET-h/d)	30.6 ± 15.3	30.0 ± 15.4	31.0 ± 15.3	30.6 ± 15.2	27.3 ± 15.8	< 0.001
Consuming meat daily (%)	15.2	13.1	9.5	19.3	40.9	< 0.001
Consuming fruit daily (%)	6.7	5.8	3.6	8.5	26.5	< 0.001
BMI (kg/m^2^)	22.9 ± 3.1	22.5 ± 3.2	22.7 ± 3.1	23.1 ± 3.1	23.5 ± 3.2	< 0.001
Sleep duration (hours)	7.6 ± 1.2	7.8 ± 1.3	7.5 ± 1.2	7.7 ± 1.1	8.0 ± 1.2	< 0.001
Prevalent hypertension (%)	44.4	43.9	45.5	43.4	42.6	< 0.001
Women with menopause (%)	53.7	61.9	56.8	49.0	47.3	< 0.001

MET: metabolic equivalent tasks; CNY: Chinese Yuan; BMI: body mass index

### Associations of fish/seafood consumption with risk of stroke

During 643,100 person-years (median 12.4 years) of follow-up, 2994 total strokes, 2213 IS, and 772 HS were identified. After adjustment for socio-demographic status, cigarette smoking, alcohol drinking, physical activity, dietary habit, sleep duration, and prevalent hypertension, in comparison with non-consumers, the adjusted HRs (95%CI) for incident total stroke risk were 1.09 (0.95–1.25) for monthly, 1.01 (0.88–1.17) for 1–3 days/week, and 0.83 (0.64–1.07) for ≥ 4 days/week (*p*-trend = 0.124). Similarly, no significant associations were observed with IS (*p*-trend = 0.191), or HS (*p*-trend = 0.518) **(**Table 2**).**

**Table 2:** Association of fish/seafood consumption frequency with risk of incident stroke

	Case, *n*	Incidence per 1000 PYs	Model 1 HR (95%CI)	Model 2 HR (95%CI)	Model 3 HR (95%CI)
Total stroke					
Never/rarely	249	5.4	1.00 (Ref)	1.00 (Ref)	1.00 (Ref)
Monthly	1481	5.2	1.10 (0.96–1.26)	1.09 (0.96–1.25)	1.09 (0.95–1.25)
1–3 days/week	1186	4.1	1.07 (0.93–1.23)	1.02 (0.89–1.18)	1.01 (0.88–1.17)
≥ 4 days/week	78	3.5	0.93 (0.72–1.20)	0.85 (0.65–1.10)	0.83 (0.64–1.07)
* p-*Trend			0.755	0.206	0.124
IS					
Never/rarely	182	3.9	1.00 (Ref)	1.00 (Ref)	1.00 (Ref)
Monthly	1100	3.9	1.13 (0.96–1.32)	1.12 (0.96–1.31)	1.11 (0.95–1.30)
1–3 days/week	868	3.0	1.08 (0.92–1.27)	1.03 (0.88–1.22)	1.01 (0.86–1.20)
≥ 4 days/week	63	2.8	1.04 (0.77–1.38)	0.93 (0.69–1.24)	0.90 (0.67–1.21)
* p-*Trend			0.983	0.321	0.191
HS					
Never/rarely	65	1.4	1.00 (Ref)	1.00 (Ref)	1.00 (Ref)
Monthly	374	1.3	1.04 (0.80–1.36)	1.04 (0.80–1.36)	1.03 (0.79–1.35)
1–3 days/week	317	1.1	1.05 (0.80–1.38)	1.02 (0.79–1.36)	1.01 (0.77–1.34)
≥ 4 days/week	16	0.7	0.72 (0.40–1.21)	0.68 (0.38–1.16)	0.67 (0.37–1.13)
* p-*Trend			0.738	0.581	0.518

Model 1: adjusted for age, sex, education attainment, income, and marital status. Model 2: additionally adjusted for cigarette smoking, alcohol drinking, meat consumption, fresh fruit consumption, physical activity, sleep duration, and BMI. Model 3: additionally adjusted for prevalent hypertension as compared to model 2. Abbreviations: HRs, hazard ratios. CI, confidence interval. Ref, reference. IS, ischemic stroke. HS, hemorrhagic stroke. PYs, person-years

### Stratified analyses

With regard to total stroke, the adjusted HR (95%CI) among men was 0.90 (0.74–1.11) for monthly, 0.83 (0.68–1.03) for 1–3 days/week, and 0.64 (0.45–0.91) for ≥ 4 days/week consumption in comparison with non-consumers (*p*-trend = 0.009) **(**Table 3**)**. The corresponding HR (95%CI) for women was 1.23 (1.03–1.48), 1.15 (0.95–1.40), and 1.02 (0.69–1.48), respectively (*p*-trend = 0.844) **(**Table 4**)**. An inverse association between fish/seafood consumption and total stroke was stronger among men than among women (*p*-interaction = 0.035). With regard to IS, the adjusted HR (95%CI) among men was 0.94 (0.74–1.20) for monthly, 0.85 (0.67–1.09) for 1–3 days/week, and 0.67 (0.44-1.00) for ≥ 4 days/week consumption in comparison with non-consumers (*p*-trend = 0.024) **(**Table 3**)**. The corresponding HR (95%CI) for women was 1.23 (0.99–1.52), 1.13 (0.91–1.41), and 1.19 (0.77–1.79), respectively (*p*-trend = 0.795) **(**Table 4**)**. An inverse association between fish/seafood consumption and IS was marginally more pronounced among men than among women (*p*-interaction = 0.062). Concerning HS, the adjusted HR (95%CI) among men was 0.78 (0.55–1.15) for monthly, 0.77 (0.54–1.14) for 1–3 days/week, and 0.57 (0.27–1.10) for ≥ 4 days/week consumption in comparison with non-consumers (*p*-trend = 0.198) **(**Table 3**)**. The corresponding HR (95%CI) for women was 1.31 (0.91–1.96), 1.29 (0.88–1.95), and 0.68 (0.23–1.62), respectively (*p*-trend = 0.790) **(**Table 4**)**.

**Table 3:** Associations of fish/seafood consumption frequency with risk of stroke among men

	Case, *n*	Incidence per 1000 PYs	Model 1	Model 2	Model 3
			HR (95%CI)	HR (95%CI)	HR (95%CI)
Total stroke					
Never/rarely	112	8.4	1.00 (Ref)	1.00 (Ref)	1.00 (Ref)
Monthly	721	6.8	0.95 (0.78–1.16)	0.92(0.76–1.14)	0.90 (0.74–1.11)
1–3 days/week	662	5.1	0.93 (0.76–1.14)	0.87 (0.71–1.07)	0.83 (0.68–1.03)
≥ 4 days/week	44	4.0	0.79 (0.55–1.11)	0.67 (0.47–0.95)	0.64 (0.45–0.91)
* p-*Trend			0.274	0.025	0.009
IS					
Never/rarely	78	5.8	1.00 (Ref)	1.00 (Ref)	1.00 (Ref)
Monthly	526	4.9	0.99 (0.78–1.26)	0.96 (0.76–1.23)	0.94 (0.74–1.20)
1–3 days/week	479	3.7	0.96 (0.76–1.23)	0.89 (0.70–1.14)	0.85 (0.67–1.09)
≥ 4 days/week	33	3.0	0.84 (0.55–1.26)	0.70 (0.46–1.05)	0.67 (0.44-1.00)
* p-*Trend			0.445	0.054	0.024
HS					
Never/rarely	34	2.5	1.00 (Ref)	1.00 (Ref)	1.00 (Ref)
Monthly	189	1.7	0.81 (0.57–1.19)	0.80 (0.56–1.17)	0.78 (0.55–1.15)
1–3 days/week	183	1.4	0.83 (0.58–1.22)	0.80 (0.56–1.17)	0.77 (0.54–1.14)
≥ 4 days/week	11	1.0	0.65 (0.31–1.25)	0.59 (0.28–1.14)	0.57 (0.27–1.10)
* p-*Trend			0.426	0.258	0.198

Model 1: hazard ratios were adjusted for age, education attainment, income, and marital status. Model 2: hazard ratios were additionally adjusted for cigarette smoking, alcohol drinking, meat consumption, fresh fruit consumption, physical activity, sleep duration, and BMI. Model 3: hazard ratios were additionally adjusted for prevalent hypertension. Abbreviations: HRs, hazard ratios. CI, confidence interval. Ref, reference. IS, ischemic stroke. HS, hemorrhagic stroke. PYs, person-years

**Table 4:** Associations of fish/seafood consumption frequency with risk of stroke among women

	Case, *n*	Incidence per 1000 PYs	Model 1	Model 2	Model 3
			HR (95%CI)	HR (95%CI)	HR (95%CI)
Total stroke					
Never/rarely	137	4.2	1.00 (Ref)	1.00 (Ref)	1.00 (Ref)
Monthly	760	4.3	1.23 (1.03–1.49)	1.23 (1.03–1.48)	1.23 (1.03–1.48)
1–3 days/week	524	3.2	1.18 (0.98–1.44)	1.15 (0.96–1.40)	1.15 (0.95–1.40)
≥ 4 days/week	34	3.1	1.08 (0.73–1.56)	1.05 (0.71–1.53)	1.02 (0.69–1.48)
*p-*Trend			0.525	0.733	0.844
IS					
Never/rarely	104	3.2	1.00 (Ref)	1.00 (Ref)	1.00 (Ref)
Monthly	574	3.2	1.24 (1.01–1.53)	1.22 (0.99–1.52)	1.23 (0.99–1.52)
1–3 days/week	389	2.4	1.17 (0.94–1.46)	1.13 (0.91–1.42)	1.13 (0.91–1.41)
≥ 4 days/week	30	2.7	1.27 (0.83–1.88)	1.22 (0.79–1.83)	1.19 (0.77–1.79)
* p-*Trend			0.480	0.696	0.795
HS					
Never/rarely	31	0.9	1.00 (Ref)	1.00 (Ref)	1.00 (Ref)
Monthly	185	1.0	1.30 (0.90–1.95)	1.31 (0.91–1.96)	1.31 (0.91–1.96)
1–3 days/week	134	0.8	1.30 (0.89–1.97)	1.30 (0.88–1.96)	1.29 (0.88–1.95)
≥ 4 days/week	5	0.4	0.70 (0.24–1.65)	0.70 (0.23–1.67)	0.68 (0.23–1.62)
* p-*Trend			0.711	0.736	0.790

Model 1: hazard ratios were adjusted for age, education attainment, income, and marital status. Model 2: hazard ratios were additionally adjusted for cigarette smoking, alcohol drinking, meat consumption, fresh fruit consumption, physical activity, sleep duration, and BMI. Model 3: hazard ratios were additionally adjusted for prevalent hypertension. HRs, hazard ratios. CI, confidence interval. Ref, reference. IS, ischemic stroke. HS, hemorrhagic stroke. PYs, person-years

### Subgroup analyses

The strength of the association between fish/seafood consumption and total stroke was consistent across subgroups defined by education level, household income, alcohol status, physical activity, meat consumption, fruit consumption, BMI, and sleep duration. However, there was a significantly stronger association among current smokers (HR = 0.84, 95% CI: 0.73–0.95) than non-current smokers (HR = 0.98, 95% CI: 0.89–1.07) (*p*-interaction = 0.046), among participants without hypertension (HR = 0.82, 95% CI: 0.71–0.95) than those with hypertension (HR = 0.97, 95% CI: 0.89–1.07) (*p*-interaction = 0.042). The magnitude of the association was marginally more pronounced among participants aged 30–49 years than those aged 50–79 years. **(**Table 5**)**

**Table 5:** Adjusted hazard ratios for incident total stroke associated with consuming fish/seafood weekly versus non-weekly by participant characteristics

	Case, *n*	HR (95%CI)	*p*-interaction
Age (years)			0.052
30–49	329	0.76 (0.61–0.95)	
50–79	2665	0.96 (0.88–1.04)	
Education level			0.587
No formal education	1718	0.95 (0.86–1.05)	
Primary or above	1276	0.91 (0.81–1.02)	
Household income (CNY)			0.171
< 35,000	1998	0.96 (0.88–1.06)	
≥ 35,000	996	0.87 (0.76–0.98)	
Smoking status			0.046
Current smokers	967	0.84 (0.73–0.95)	
Non-current smokers	2027	0.98 (0.89–1.07)	
Alcohol status			0.807
Current drinkers	642	0.92 (0.79–1.07)	
Non-current drinkers	2352	0.94 (0.86–1.02)	
Physical activities (MET-h/d)			0.717
< 30	1960	0.94 (0.86–1.03)	
≥ 30	1034	0.92 (0.81–1.04)	
Meat consumption			0.952
Daily	402	0.94 (0.76–1.15)	
Non-daily	2592	0.94 (0.87–1.02)	
Fruit consumption			0.626
Daily	150	0.86 (0.61–1.22)	
Non-daily	2844	0.94 (0.87–1.01)	
Body mass index (kg/m^2^)			0.342
< 25	2197	0.92 (0.84-1.00)	
≥ 25	797	0.99 (0.86–1.14)	
Sleep duration (hours/day)			0.845
< 7.6	1365	0.94 (0.84–1.05)	
≥ 7.6	1629	0.93 (0.84–1.02)	
Prevalent hypertension			0.042
Yes	2201	0.97 (0.89–1.06)	
No	793	0.82 (0.71–0.95)	

Hazard ratios were adjusted for age, sex, education level, household income, marital status, cigarette smoking, alcohol drinking, meat consumption, fresh fruit consumption, physical activity, sleep duration, BMI, and prevalent hypertension. HRs, hazard ratios. CI, confidence interval. CNY, Chinese Yuan.‌ MET, metabolic equivalent tasks

With regard to IS, the strength of the association was consistent across subgroups defined by age, education level, household income, alcohol status, physical activity, meat consumption, fruit consumption, BMI, sleep duration, and prevalent hypertension. The magnitude of the association was marginally more pronounced among current smokers (HR = 0.83, 95% CI: 0.71–0.96) than non-current smokers (HR = 0.97, 95% CI: 0.87–1.07) (*p*-interaction = 0.094). (Additional file 1: Table S1)

### Sensitivity analyses

In sensitivity analyses, the associations did not change appreciably after further adjustment for menopause status among women (Additional file 1: Table S2). In the competing risks analysis, the main outcomes were largely consistent with those from the primary analysis (Additional file 1: Table S3). Association between fish intake and IS was attenuated, and a marginal significance was observed among men (*p*-trend = 0.084). After excluding participants with less than 2 years of follow-up (Additional file 1: Table S4), stroke results were largely unchanged.

## Discussion

The present study sheds light on the association of fish/seafood consumption with the risk of stroke and its subtypes. Among all participants, fish/seafood consumption was not associated with stroke or its subtypes. Among men, a significant inverse association was observed with total stroke and IS (but not with HS), whereas among women, no significant association was observed with stroke and its subtypes.

Coherent with previous studies, participants who more frequently consumed fish/seafood were more likely to be younger [17, 18], men [17, 18], having a higher level of formal education [16, 17], wealthy [16, 18], current smokers [17], current drinkers [16, 17], to consume meat and fruit more frequently [16, 17, 26], to have a higher BMI [16], and less likely to have hypertension [18, 27]. Chinese Dietary Guidelines (2022) recommended consuming fish at least twice per week or 300–500 g per week for adults [28]. The proportion of participants consuming fish at least once per week in the present study was 48.2%, far behind the recommendation of guidelines. These findings underscore the need for tailored nutrition education to bridge the intake gap.

### Associations of fish/seafood consumption with stroke

Evidence on the association of fish/seafood consumption and stroke from published papers was contradictory. For example, one early meta-analysis of 0.38 million participants from fifteen prospective studies (7 in the US, 4 in Europe, 3 in Japan, and 1 in China) indicated that a 3-serving/week increase in fish intake was associated with lower total stroke risk (RR = 0.94, 95%CI: 0.89–0.99) and IS risk (RR = 0.90, 95%CI: 0.84–0.97), but not HS (RR = 0.90, 95%CI: 0.76–1.06) [29]. Another meta-analysis of ten cohort studies confirmed this IS benefit (RR = 0.81, 95%CI: 0.70–0.94), and found no association with HS (RR = 1.01, 95%CI: 0.76–1.34) [30]. Contrary to the two meta-analyses above, a third meta-analysis of 13 prospective studies reported HS protection (HR = 0.88, 95%CI: 0.80–0.96), but no IS effect (15 prospective studies included) (HR = 0.96, 95%CI: 0.89–1.03) [31]. The health effects of fish consumption on stroke varied by geographic regions, and significant effects were observed in the Asia-Pacific region, but not in Europe or North America [31]. Variances in racial groups, lifestyles, dietary patterns, cooking methods, and contaminant levels in fish/seafood may account for discrepancies between studies [32]. In terms of Chinese populations, one prospective cohort study involving‌ over 57,000 adults aged 20–74 years without history of stroke, with a median follow-up of 4.6 years, documented that compared with fish intake of less than 300 g/week, 300–450 g/week fish intake reduced incident IS risk (HR = 0.70, 95%CI: 0.57–0.88), but not incident HS [17], compatible with our findings. However, another cohort study comprising 95,800 participants with more than 703,000 person-years of follow-up observed that compared with the first quartile of fish intake, HR (95%CI) for the fourth quartile of fish intake was 0.68 (0.59–0.78) for incident total stroke, 0.70 (0.59–0.83) for incident IS and 0.72 (0.57–0.93) for incident HS, respectively [18], and the results of HS were inconsistent with our results. A Guangzhou cohort study of more than 18,000 participants aged ≥ 50 years without CVD at baseline, with a mean follow-up of 11.4 years, demonstrated that fish consumption was not significantly associated with any of total stroke, IS, and HS mortality [16]. The results of total stroke and IS were contrary to the present study. One of the possible explanations of the discrepancies may be the insufficient statistical power due to the limited death cases in Guangzhou study [16].

*A* meta-analysis of prospective studies indicated that an inverse association was more pronounced in women (HR = 0.83, 95%CI: 0.75–0.92) than in men (HR = 0.97, 95%CI: 0.84–1.11) [31]. In contrast, a nationally representative survey of more than 30,000 Korean adults aged 19–64 years indicated that compared with non-consumers, odds ratios (ORs) (95%CI) for stroke among participants who consumed fish 0 < to < 1/2 servings per day, 1/2 < to < 1 servings per day, and ≥ 1 servings per day was 0.72 (0.39–1.34), 0.94 (0.43–2.03), and 0.47 (0.23–0.97), respectively in men, but no inverse association was observed in women [33]. In the present study, the association of fish consumption with reduced risk of total stroke and IS was more pronounced in men, which was compatible with Shanghai prospective cohort study [17]. Interestingly, the beneficial association between types of diets and cardiovascular outcomes in the UK Biobank was stronger in men than in women [34]. A similar sex difference was observed in the association between fish intake and type 2 diabetes among Japanese adults [32]. The reason behind the sex-difference association was unclear. It may be partly explained by hormonal factors and lifestyle differences [17]. Future studies are needed to verify this association and further ascertain the reason behind the sex difference observed. In addition, women who consumed fish/seafood monthly had a higher total stroke risk than those who never/rarely consumed it in the current study. However, such a positive association was not observed with IS, which was the main type of total stroke. The reason behind this phenomenon is unclear.

It is noteworthy that the association between fish/seafood consumption and total stroke was stronger among current smokers than non-current smokers. China, as the world’s largest producer and consumer of tobacco, has over 300 million smokers, nearly one-third of the world’s total [35]. Given that smoking constitutes a major risk factor for CVD, our findings suggest that smokers should maintain adequate fish/seafood intake to potentially reduce stroke risk. In addition, the association between fish/seafood intake and total stroke risk was more pronounced among participants without hypertension than those with hypertension, indicating the importance of incorporating fish/seafood consumption into nutritional intervention strategies for individuals with normal blood pressure and prehypertensive individuals.

### Possible mechanisms

There are several possible mechanisms underlying the inverse association between fish/seafood consumption and IS risk. Fish/seafood is abundant in long-chain omega-3 PUFAs. PUFAs may have a beneficial effect on IS through several possible mechanisms. First, PUFAs may reduce plasma triglyceride concentrations [36, 37]. Second, blood pressure is one of the most important risk factors of stroke, and PUFAs may lower the systolic blood pressure level [38]. Third, PUFAs could promote the synthesis of prostaglandin I3, an antithrombotic agent [39]. Fourth, long-chain omega-3 PUFAs may reduce stroke via their anti-atherogenic and neuroprotective effects [40–42]. Additionally, multiple bioactive nutrients, such as essential amino acids, fat-soluble vitamins, minerals, and other fatty acids in fish/seafood, may provide health benefits [43–45]. For example, selenium and calcium in fish could potentially alleviate oxidative stress and inflammation in patients with CVD [46], while vitamin D could potentially regulate blood pressure, inhibit inflammation, and inhibit vascular smooth muscle proliferation and vascular calcification [47].

Several limitations need to be acknowledged. First, the assessment of fish/seafood consumption was qualitative, without quantitative data. Fish/seafood consumption was assessed merely using baseline data, which might result in an underestimation of risk associations due to regression dilution bias [48]. Second, information on fish/seafood cooking methods (e.g., frying, steaming) was not collected in the CKB questionnaire. Fried cooking not only causes omega-3 loss in fish, but also generates harmful substances [49], thereby increasing the risk of stroke [27], whereas raw fish will not [9]. Fish is commonly steamed in China, but is often fried in the US [17]. Third, details on fish types (e.g., salmon vs. tilapia) and sources (wild vs. farmed) were not specified, despite known variations in nutrient profiles and contaminant levels‌ [50]. Fourth, due to the limited number of cases, the association of fish/seafood consumption with stroke should be interpreted with caution, especially for HS. Fifth, only one rural region’s data was used in the present study. Hence, the results should be interpreted cautiously when generalizing to other populations. In addition, the results exclude individuals who did not participate in the prospective study, which may lead to selection bias. Sixth, the observational nature of the study does not allow a conclusion of a causal relationship between fish/seafood consumption and stroke. Seventh, only baseline dietary data, without repeated measurements, may not adequately capture potential changes in dietary exposure during long follow-up periods. Eighth, although several established and potential risk factors were adjusted for, residual confounding by other unmeasured important factors, such as total energy intake, cannot be ruled out.

## Conclusions

In summary, among all participants, fish/seafood consumption was not associated with total stroke or its subtypes. Among men, a significant inverse association was observed with total stroke and IS (but not with HS). In contrast, among women, no significant association was observed with total stroke or its subtypes. Given the low baseline fish/seafood consumption observed in the present study, our findings support the recommendation of consuming non-fried fish at least twice per week, and encourage Chinese adults to consume fish moderately. Future studies need to validate these subtype- and sex-specific associations in larger cohort studies or randomized controlled trials.

## Electronic Supplementary Material

Below is the link to the electronic supplementary material.

Supplementary Material 1.

## Data Availability

Details of how to access China Kadoorie Biobank data and details of the data release schedule are available from www.ckbiobank.org/site/Data+Access.

## Abbreviations

IS: Ischemic stroke
HS: Hemorrhagic stroke
CVD: Cardiovascualr disease
HRs: Hazard ratios
CI: Confidence interval
GBD: Global burden disease
CKB: China Kadoorie Biobank
CNY: Chinese Yuan
PUFAs: Polyunsaturated fatty acids
MET: Metabolic equivalent task
BMI: Body mass index
SBP: Systolic blood pressure
DBP: Diastolic blood pressure
RBG: Random blood glucose
SD: Standard deviation
OR_S_: Odds ratios
RR: Relative ratio
FFQ: Food frequency questionnaire
Ref: Reference
US: United States
UK: United Kingdom
