# Supplementary material for: Frequency of fish/seafood consumption and risk of stroke: a prospective cohort study in Zhejiang, China
Source: Nutr Metab (Lond). 2026 May 11;23:77. doi: 10.1186/s12986-026-01136-x (PMC13335027; doi:10.1186/s12986-026-01136-x)
Supplement: Supplementary file 1 — Supplementary Material 1. [file 12986_2026_1136_MOESM1_ESM.docx]

**`Table S1. Adjusted hazard ratios for incident ischemic stroke associated with consuming fish/seafood weekly versus non-weekly by participant characteristics**

|  | **Case, n** | **HR (95%CI)** | **P for interaction** |
| --- | --- | --- | --- |
| Age (years) |  |  | 0.368 |
| 30-49 | 223 | 0.82 (0.63-1.07) |  |
| 50-79 | 1990 | 0.94 (0.85-1.03) |  |
| Education level |  |  | 0.675 |
| No formal education | 1284 | 0.94 (0.83-1.04) |  |
| Primary or above | 929 | 0.90 (0.79-1.03) |  |
| Household income (CNY) |  |  | 0.194 |
| < 35,000 | 1469 | 0.96 (0.86-1.07) |  |
| ≥ 35,000 | 744 | 0.85 (0.74-0.99) |  |
| Smoking status |  |  | 0.094 |
| Current smokers | 691 | 0.83 (0.71-0.96) |  |
| Non-current smokers | 1522 | 0.97 (0.87-1.07) |  |
| Alcohol status |  |  | 0.532 |
| Current drinkers | 457 | 0.88 (0.73-1.06) |  |
| Non-current drinkers | 1756 | 0.94 (0.85-1.04) |  |
| Physical activities (MET-h/d) |  |  | 0.397 |
| < 30 | 1465 | 0.95 (0.85-1.06) |  |
| ≥ 30 | 748 | 0.88 (0.76-1.02) |  |
| Meat consumption |  |  | 0.899 |
| Daily | 299 | 0.94 (0.75-1.20) |  |
| Non-daily | 1914 | 0.93 (0.85-1.02) |  |
| Fruit consumption |  |  | 0.517 |
| Daily | 113 | 1.05 (0.71-1.61) |  |
| Non-daily | 2100 | 0.92 (0.84-1.00) |  |
| Body mass index (kg/m^2^) |  |  | 0.523 |
| < 25 | 1594 | 0.91 (0.82-1.01) |  |
| ≥ 25 | 619 | 0.97 (0.83-1.14) |  |
| Sleep duration (hours/day) |  |  | 0.976 |
| < 7.6 | 1003 | 0.92 (0.81-1.05) |  |
| ≥ 7.6 | 1210 | 0.92 (0.82-1.04) |  |
| Prevalent hypertension |  |  | 0.110 |
| Yes | 1634 | 0.96 (0.87-1.06) |  |
| No | 579 | 0.82 (0.69-0.97) |  |

Hazard ratios were adjusted for age, sex, education level, household income, marital status, cigarette smoking, alcohol drinking, meat consumption, fresh fruit consumption, physical activity, sleep duration, BMI, and prevalent hypertension. Abbreviations: HRs, hazard ratios. CI, confidence interval. CNY, Chinese Yuan.‌ MET, metabolic equivalent tasks.

**Table S2. Association between fish/seafood consumption frequency and risk of stroke among women after further adjustment for menopause status**

|  |  | Total stroke |  |  |  | IS |  |  |  | HS |  |
| --- | --- | --- | --- | --- | --- | --- | --- | --- | --- | --- | --- |
|  | Case, n | Incidence per 1000 PYs | HR (95%CI) |  | Case, n | Incidence per 1000 PYs | HR (95%CI) |  | Case, n | Incidence per 1000 PYs | HR (95%CI) |
| Never/rarely | 137 | 4.2 | 1.00 (Ref) |  | 104 | 3.2 | 1.00 (Ref) |  | 31 | 0.9 | 1.00 (Ref) |
| Monthly | 760 | 4.3 | 1.23 (1.03-1.48) |  | 574 | 3.2 | 1.23 (1.00-1.52) |  | 185 | 1.0 | 1.32 (0.91-1.99) |
| 1-3 days/week | 524 | 3.2 | 1.15 (0.95-1.40) |  | 389 | 2.4 | 1.13 (0.91-1.41) |  | 134 | 0.8 | 1.36 (0.92-2.08) |
| ≥ 4 days/week | 34 | 3.1 | 1.02 (0.69-1.49) |  | 30 | 2.7 | 1.16 (0.76-1.74) |  | 5 | 0.4 | 0.62 (0.18-1.59) |
| *p-*Trend |  |  | 0.846 |  |  |  | 0.816 |  |  |  | 0.546 |


Hazard ratios were adjusted for age, education level, household income, marital status, cigarette smoking, alcohol drinking, meat consumption, fresh fruit consumption, physical activity, sleep duration, BMI, prevalent hypertension, and menopause status. HRs, hazard ratios. CI, confidence interval. Ref, reference. IS, ischemic stroke. HS, hemorrhagic stroke. PYs, person-years.

**Table S3. Association between fish/seafood consumption frequency and risk of stroke in competing risk analysis**

|  |  | Total stroke |  |  |  | IS |  |  |  | HS |  |
| --- | --- | --- | --- | --- | --- | --- | --- | --- | --- | --- | --- |
|  | Case, n | Incidence per 1000 PYs | HR (95%CI) |  | Case, n | Incidence per 1000 PYs | HR (95%CI) |  | Case, n | Incidence per 1000 PYs | HR (95%CI) |
| Total |  |  |  |  |  |  |  |  |  |  |  |
| Never/rarely | 249 | 5.4 | 1.00 (Ref) |  | 182 | 3.9 | 1.00 (Ref) |  | 65 | 1.4 | 1.00 (Ref) |
| Monthly | 1481 | 5.2 | 1.07 (0.94-1.23) |  | 1100 | 3.9 | 1.10 (0.94-1.29) |  | 374 | 1.3 | 1.00 (0.75-1.33) |
| 1-3 days/week | 1186 | 4.1 | 1.01 (0.88-1.17) |  | 868 | 3.0 | 1.02 (0.87-1.21) |  | 317 | 1.1 | 1.01 (0.75-1.36) |
| ≥ 4 days/week | 78 | 3.5 | 0.84 (0.65-1.09) |  | 63 | 2.8 | 0.93 (0.69-1.26) |  | 16 | 0.7 | 0.63 (0.34-1.18) |
| *p-*Trend |  |  | 0.195 |  |  |  | 0.320 |  |  |  | 0.568 |
| Men ^a^ |  |  |  |  |  |  |  |  |  |  |  |
| Never/rarely | 112 | 8.4 | 1.00 (Ref) |  | 78 | 5.8 | 1.00 (Ref) |  | 34 | 2.5 | 1.00 (Ref) |
| Monthly | 721 | 6.8 | 0.89 (0.73-1.10) |  | 526 | 4.9 | 0.97 (0.76-1.23) |  | 189 | 1.7 | 0.69 (0.48-1.01) |
| 1-3 days/week | 662 | 5.1 | 0.83 (0.68-1.03) |  | 479 | 3.7 | 0.90 (0.70-1.15) |  | 183 | 1.4 | 0.68 (0.46-1.00) |
| ≥ 4 days/week | 44 | 4.0 | 0.65 (0.45-0.93) |  | 33 | 3.0 | 0.72 (0.47-1.11) |  | 11 | 1.0 | 0.53 (0.25-1.09) |
| *p*-Trend |  |  | 0.014 |  |  |  | 0.084 |  |  |  | 0.122 |
| Women ^a^ |  |  |  |  |  |  |  |  |  |  |  |
| Never/rarely | 137 | 4.2 | 1.00 (Ref) |  | 104 | 3.2 | 1.00 (Ref) |  | 31 | 0.9 | 1.00 (Ref) |
| Monthly | 760 | 4.3 | 1.22 (1.02-1.47) |  | 574 | 3.2 | 1.22 (0.98-1.50) |  | 185 | 1.0 | 1.45 (0.93-2.24) |
| 1-3 days/week | 524 | 3.2 | 1.16 (0.96-1.41) |  | 389 | 2.4 | 1.13 (0.91-1.42) |  | 134 | 0.8 | 1.51 (0.96-2.38) |
| ≥ 4 days/week | 34 | 3.1 | 1.05 (0.72-1.55) |  | 30 | 2.7 | 1.23 (0.81-1.87) |  | 5 | 0.4 | 0.58 (0.17-1.99) |
| *p-*Trend |  |  | 0.653 |  |  |  | 0.635 |  |  |  | 0.392 |


Hazard ratios were adjusted for age, sex, education level, household income, marital status, cigarette smoking, alcohol drinking, meat consumption, fresh fruit consumption, physical activity, sleep duration, BMI, and prevalent hypertension. Abbreviations: HRs, hazard ratios. CI, confidence interval. Ref, reference. IS, ischemic stroke. HS, hemorrhagic stroke. PYs, person-years.

a: without adjustment for sex.

**Table S4. Association between fish/seafood consumption frequency and risk of stroke after excluding participants with less than 2 years of follow-up**

|  |  | Total stroke |  |  |  | IS |  |  |  | HS |  |
| --- | --- | --- | --- | --- | --- | --- | --- | --- | --- | --- | --- |
|  | Case, n | Incidence per 1000 PYs | HR (95%CI) |  | Case, n | Incidence per 1000 PYs | HR (95%CI) |  | Case, n | Incidence per 1000 PYs | HR (95%CI) |
| Total |  |  |  |  |  |  |  |  |  |  |  |
| Never/rarely | 219 | 4.8 | 1.00 (Ref) |  | 165 | 3.6 | 1.00 (Ref) |  | 52 | 1.1 | 1.00 (Ref) |
| Monthly | 1330 | 4.7 | 1.28 (1.06-1.57) |  | 1001 | 3.5 | 1.13 (0.96-1.34) |  | 322 | 1.1 | 1.14 (0.85-1.54) |
| 1-3 days/week | 1086 | 3.7 | 1.20 (0.98-1.47) |  | 800 | 2.7 | 1.03 (0.87-1.23) |  | 287 | 1.0 | 1.16 (0.86-1.58) |
| ≥ 4 days/week | 71 | 3.2 | 1.04 (0.69-1.54) |  | 60 | 2.7 | 0.94 (0.69-1.26) |  | 12 | 0.5 | 0.62 (0.31-1.12) |
| *p-*Trend |  |  | 0.265 |  |  |  | 0.300 |  |  |  | 0.904 |
| Men ^a^ |  |  |  |  |  |  |  |  |  |  |  |
| Never/rarely | 97 | 7.3 | 1.00 (Ref) |  | 70 | 5.3 | 1.00 (Ref) |  | 26 | 1.9 | 1.00 (Ref) |
| Monthly | 638 | 6.0 | 0.94 (0.76-1.17) |  | 475 | 4.4 | 0.95 (0.75-1.24) |  | 157 | 1.4 | 0.87 (0.59-1.36) |
| 1-3 days/week | 604 | 4.6 | 0.88 (0.71-1.10) |  | 439 | 3.4 | 0.87 (0.67-1.13) |  | 166 | 1.3 | 0.92 (0.62-1.44) |
| ≥ 4 days/week | 40 | 3.6 | 0.66 (0.45-0.95) |  | 32 | 2.9 | 0.71 (0.46-1.08) |  | 8 | 0.7 | 0.52 (0.22-1.11) |
| *p*-Trend |  |  | 0.034 |  |  |  | 0.049 |  |  |  | 0.523 |
| Women ^a^ |  |  |  |  |  |  |  |  |  |  |  |
| Never/rarely | 122 | 3.7 | 1.00 (Ref) |  | 95 | 2.9 | 1.00 (Ref) |  | 26 | 0.8 | 1.00 (Ref) |
| Monthly | 692 | 3.9 | 1.28 (1.06-1.57) |  | 526 | 3.0 | 1.25 (1.01-1.57) |  | 165 | 0.9 | 1.42 (0.96-2.21) |
| 1-3 days/week | 482 | 3.0 | 1.20 (0.98-1.47) |  | 361 | 2.2 | 1.15 (0.92-1.46) |  | 121 | 0.7 | 1.40 (0.93-2.20) |
| ≥ 4 days/week | 31 | 2.8 | 1.04 (0.68-1.54) |  | 28 | 2.5 | 1.21 (0.77-1.84) |  | 4 | 0.4 | 0.64 (0.19-1.67) |
| *p-*Trend |  |  | 0.670 |  |  |  | 0.706 |  |  |  | 0.673 |


Hazard ratios were adjusted for age, sex, education level, household income, marital status, cigarette smoking, alcohol drinking, meat consumption, fresh fruit consumption, physical activity, sleep duration, BMI, and prevalent hypertension. HRs, hazard ratios. CI, confidence interval. Ref, reference. IS, ischemic stroke. HS, hemorrhagic stroke. PYs, person-years.

a: without adjustment for sex.
